# *Sorbus commixta* Fruit Extract Suppresses Lipopolysaccharide-Induced Neuroinflammation in BV-2 Microglia Cells via the MAPK and NF-*κ*B Signaling Pathways

**DOI:** 10.3390/molecules29235592

**Published:** 2024-11-26

**Authors:** Yon-Suk Kim, Jin-Hwa Jung, Ki-Tae Kim

**Affiliations:** 1 Department of Biotechnology, College of Biomedical and Health Science, Research Institute of Inflammatory Diseases, Konkuk University, Chungju 27478, Republic of Korea; 2 Department of Occupational Therapy, Semyung University, Jecheon 27136, Republic of Korea; 3 Department of Korean Medicine, College of Korean Medicine, Semyung University, Jecheon 27136, Republic of Korea

**Keywords:** *Sorbus commixta* Hedl, fruit, anti-neuroinflammatory activity, BV-2 cells, mitogen-activated protein kinase

## Abstract

*Sorbus commixta* Hedl. is a traditional medicinal plant in Korea, China, and Japan with known antioxidative, anti-inflammatory, anti-atherogenic, and anti-melanin activities. However, its anti-neuroinflammatory effects remain largely unknown. In this study, we investigated the inhibitory effects of *S. commixta* fruit extracts on lipopolysaccharide-stimulated pro-inflammatory factors in BV-2 microglia. We compared the anti-neuroinflammatory activity of *S. commixta* fruit water extract (SFW) and 70% ethanol extract using a nitric oxide assay. Our data indicated that the SFW (25–100 μg/mL) treatment significantly inhibited excessive nitric oxide production in lipopolysaccharide-stimulated BV-2 microglia compared to the 70% ethanol extract. It also attenuated the expression of inducible nitric oxide synthase, cyclooxygenase-2, and pro-inflammatory cytokines such as interleukin-6 and tumor necrosis factor α. Moreover, SFW exhibited its anti-inflammatory properties by downregulating the expression of factors involved in the extracellular signal-regulated kinase, c-Jun N-terminal kinase, and p38 mitogen-activated protein kinase pathways and by suppressing nuclear factor kappa B. Caffeic acid was identified as a primary component of SFW showing anti-neuroinflammatory activity. These findings suggest that SFW may offer substantial therapeutic potential for the treatment of neurodegenerative diseases involving microglia activation.

## 1. Introduction

Microglia are macrophages in the brain that play a vital role in the inflammatory processes in the central nervous system (CNS). Microglial activity increases excessively upon exposure to stress signals, such as those from damaged nerve cells, the accumulation of abnormally folded proteins, external stimuli, or pathogen invasion, causing excessive neuroinflammatory responses and damaging nerve cells, which can lead to neurodegenerative diseases. Microglial activation causes neuroinflammation, synaptic damage, and neuronal cell death, indicating a close association between neuroinflammation and neurodegenerative diseases [1].

Microglia are activated under pathological conditions in the brain, involving the release of pro-inflammatory factors, including nitric oxide (NO), prostaglandins, and pro-inflammatory cytokines such as tumor necrosis factor α (TNF-*α*), interleukin (IL)-1*β*, and interleukin-6 (IL-6) [2]. The overproduction of these inflammatory mediators results in a range of serious neurodegenerative diseases, such as Alzheimer’s disease, cerebral ischemia, multiple sclerosis, trauma, and Parkinson’s disease [3].

Nuclear factor kappa B (NF-*κ*B) increases the production of pro-inflammatory factors such as IL-6 and TNF-*α* by regulating the expression of several inflammatory response genes [4]. In addition, NF-*κ*B is involved in the induction of inducible nitric oxide synthase (iNOS) and cyclooxygenase-2 (COX-2) expression [5]. Mitogen-activated protein kinase (MAPK) has been shown to play an important role in regulating the inflammatory process in activated microglial cells [6].

Lipopolysaccharide (LPS), a bacterial endotoxin, stimulates the differentiation of microglia into the M1 type by binding to receptors and activating inflammatory and oxidative pathways [7]. Toll-like receptor 4 (TLR4), a pattern recognition receptor for LPS, is widely expressed in the body, and when LPS binds to TLR4, an innate inflammatory response is triggered in the body, independent of the involvement of immune cells [8]. TLR4 signaling has been divided into MyD88-dependent and MyD88-independent (TRIF-dependent) pathways. The MyD88-dependent pathway is responsible for the expression of inflammatory cytokines, whereas the MyD88-independent pathway mediates the induction of type I interferon and interferon-inducible genes [9]. Also, MyD88 activates NF-*κ*B and MAPK signaling pathways, which induce the production of inflammatory cytokines [9]. LPS activates members of the MAPK family, such as p38, JNK, and ERK, in microglia, thereby producing inflammatory factors. Therefore, suppressing the NF-*κ*B and MAPK pathways may serve as an effective strategy to prevent the progression of various neurodegenerative diseases related to inflammation and microglial activation.

*Sorbus commixta* Hedl. (Rosaceae) is a well-known traditional medicinal plant in Korea, China, and Japan. Its stems, leaves, and fruits have been used in folk medicine for a variety of symptoms. This plant has been prescribed for various inflammatory symptoms such as asthma, bronchitis, gastritis, and edema [10]. Furthermore, the fruit of *S. commixta* has been used for the treatment of gastrointestinal disorders [11,12]. *Sorbus commixta* extracts exhibit a range of biological effects, such as anti-diabetic, diuretic, anti-melanoma, anti-inflammatory, antioxidative, anti-atherogenic, vasorelaxant, and anti-lipid peroxidation activities [13,14,15,16,17,18].

*Sorbus commixta* has been traditionally used as a folk medicine. However, its efficacy has only recently been verified, and research is ongoing to explore its potential as a natural material. Phytochemical analysis of *S. commixta* fruits has revealed the presence of several active ingredients, including rutin, isoquercitrin, caffeoylquinic acid, dicaffeoylquinic acid, neosakuranin, chlorogenic acid, neochlorogenic acid, carotenoids, and ascorbic acid [19,20,21]. The fruit of *S. commixta*, which contains these active ingredients, might be expected to have anti-neuroinflammatory effects; however, not much research has been conducted in this area. Therefore, in this study, we aimed to confirm the anti-neuroinflammatory effects of *S. commixta* fruit extract.

In this study, we investigated the efficacy and regulatory mechanisms of *S. commixta* fruit extract on neuroinflammation by inducing LPS-induced microglia activation using BV-2 cells, which are the microglia cells that constitute the cerebral neurovascular system.

We hypothesized that LPS-induced neuroinflammation occurs via TLR4 activation and that *S. commixta* fruit extract can suppress neuroinflammation by inhibiting MAPK and NF-*κ*B phosphorylation. We also aimed to identify the main effective components of the *S. commixta* fruit extract with anti-inflammatory properties.

## 2. Results

### 2.1. Effects of S. commixta Fruit Water Extract (SFW) and Ethanol Extract (SFE) on NO Production and Cytotoxicity

**Figure 1:**
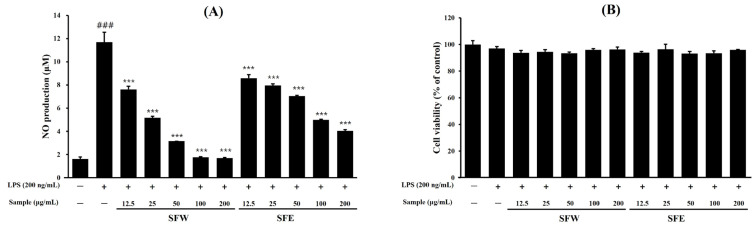
Effects of SFW and SFE on NO production (**A**) and cell viabilities (**B**) in LPS-induced BV-2 cells. BV-2 cells (2 × 10^5^ cells/mL) were treated with various concentrations of SFW (12.5–200 μg/mL) for 1 h and treated with LPS (200 ng/mL) for 24 h. Controls were samples without LPS or SFW treatment. (**A**) NO production in the culture medium was determined using the Griess reagent and a standard curve using NaNO_2_. (**B**) Cell viability was assessed by the MTT assay, and the results are expressed as the percentage of control cells. Data are presented as mean ± SD from three independent experiments. ^###^ *p* < 0.001 versus control. *** *p* < 0.001 versus LPS.

The Griess assay results showed that LPS stimulation significantly increased NO production in BV-2 cells (Figure 1A). However, when pretreated with SFW and SFE, both samples dose-dependently inhibited NO production, and the NO inhibitory effect of SFW was particularly superior to that of SFE at 25 μg/mL–200 μg/mL (Figure 1A). The cytotoxic effect of SFW and SFE on BV-2 cells was detected using an MTT assay. The results showed that there was no effect on the cytotoxicity of the SFW-treated groups up to 200 μg/mL (Figure 1B), indicating that the inhibition of NO production was not significantly affected by the cytotoxicity of SFW. Therefore, subsequent experiments were conducted using only SFW, which demonstrated superior efficacy in inhibiting NO production.

### 2.2. Effects of SFW on Pro-Inflammatory Cytokines

**Figure 2:**
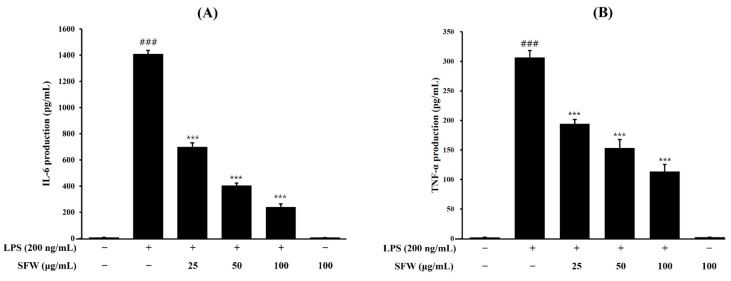
Effects of pro-inflammatory cytokines IL-6 and TNF-*α* in LPS-induced BV-2 cells (**A**,**B**). BV-2 cells were pretreated with SFW for 1 h and stimulated with LPS (200 ng/mL) for 18 h (**A**) or 6 h (**B**), and then IL-6 and TNF-*α* levels were determined using ELISA. Data are presented as mean ± SD from three independent experiments. ^###^ *p* < 0.001 versus control. *** *p* < 0.001 versus LPS.

The effects of SFW on LPS-induced IL-6 and TNF-*α* expression in BV-2 cells were analyzed using an enzyme-linked immunosorbent assay (ELISA). The results showed that the concentrations of IL-6 (Figure 2A) and TNF-*α* (Figure 2B) in the cultures significantly increased after LPS stimulation, and pretreatment with SFW decreased the production of IL-6 and TNF-*α* in a dose-dependent manner. These results show that pretreatment with SFW significantly suppresses the production of LPS-stimulated pro-inflammatory factors.

### 2.3. Effects of SFW on iNOS and COX-2 Protein Expression

**Figure 3:**
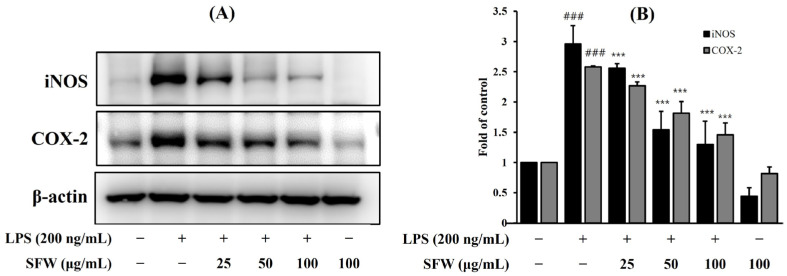
Effect of SFW on LPS-induced iNOS and COX-2 protein expressions in BV-2 cells (**A**,**B**). BV-2 cells were pretreated with SFW for 1 h, stimulated with LPS (200 ng/mL) for 18 h, and examined by Western blotting. Data are presented as mean ± SD from three independent experiments. β-actin was used as an internal control. ^###^ *p* < 0.001 versus control. *** *p* < 0.001 versus LPS.

To investigate whether the anti-inflammatory effect of SFW was associated with decreases in iNOS and COX-2 expression, we examined protein levels through Western blotting. The LPS-induced upregulations of iNOS and COX-2 proteins were attenuated dose-dependently by pretreatment with SFW (Figure 3A,B).

### 2.4. Effects of SFW on MAPK Protein Expression

**Figure 4:**
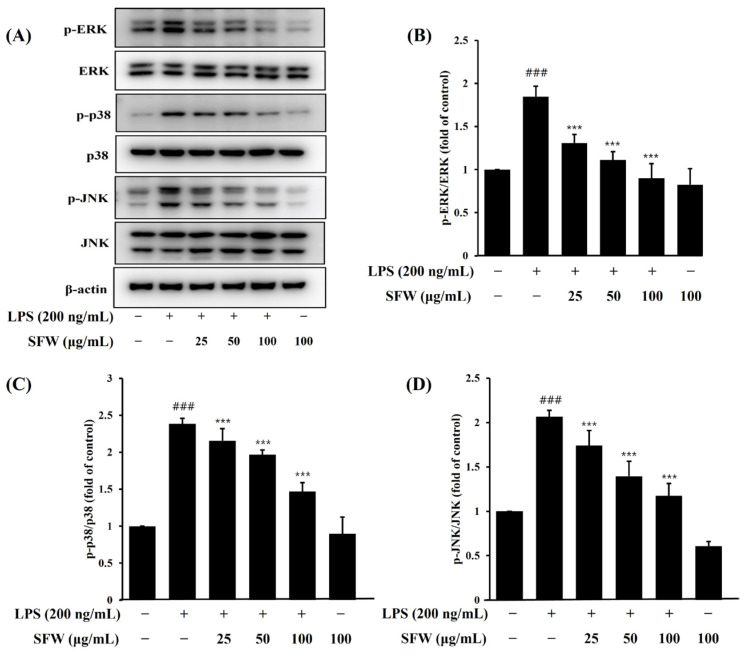
Effect of SFW on LPS-induced MAPK activation in BV-2 cells (**A**–**D**). BV-2 cells were pretreated with SFW (50–100 μg/mL) for 1 h prior to stimulation with LPS (200 ng/mL) for 30 min. Total protein (15 μg) was subjected to 12% SDS-PAGE, followed by Western blotting using anti-ERK, anti-p38, and anti-JNK. Results are representative of those obtained from three independent experiments. ^###^ *p* < 0.001 versus control. *** *p* < 0.001 versus LPS.

To investigate whether SFW regulates the MAPK pathway, we determined the effects of SFW on the LPS-stimulated expression of MAPKs (ERK, JNK, and p38) in BV-2 cells by Western blotting. As shown in Figure 4, the expressions of non-phosphorylated MAPKs (JNK, ERK and p38) were not affected by LPS or LPS with SFW. Since we confirmed through previous experiments that the expression of MAPKs significantly increased when LPS was treated for 30 min in BV-2 cells, the samples were pretreated for 1 h and then treated with LPS for 30 min [22]. Similarly to the results of our previous experiment, the LPS treatment significantly increased the phosphorylation of JNK, ERK, and p38 compared to the control group, and the SFW pretreatment significantly decreased the phosphorylation of ERK, JNK, and p38 compared with the LPS group. These results confirmed that SFW affects the regulation of the MAPK pathway.

### 2.5. Effects of SFW on Phosphorylation of IĸBα and Nuclear Translocation of NF-ĸB

**Figure 5:**
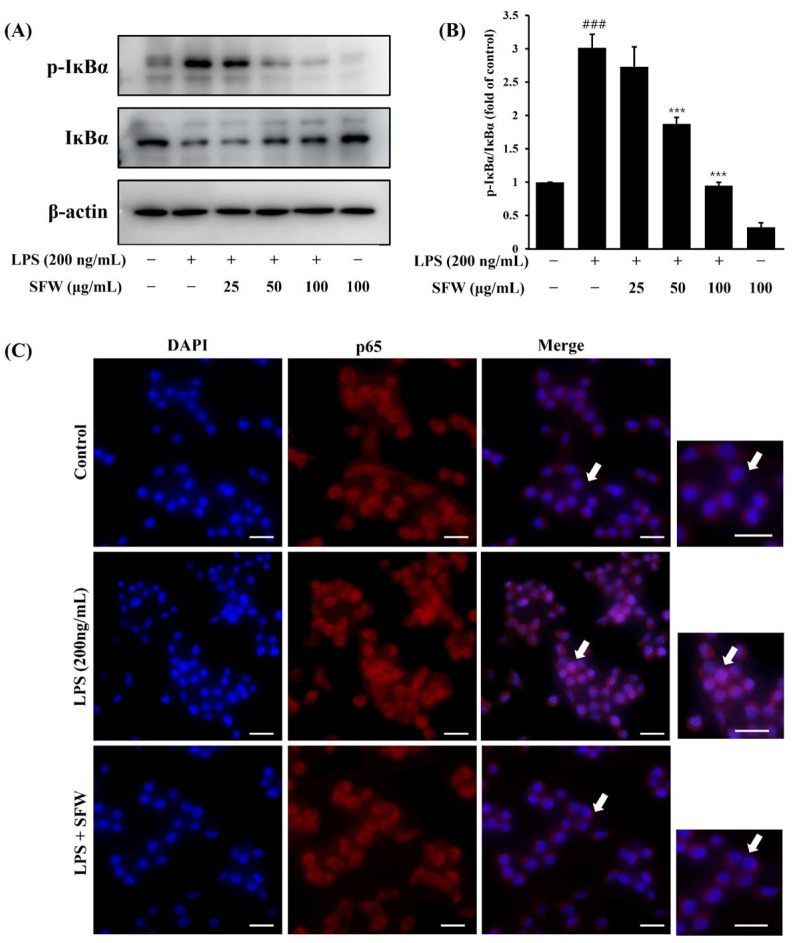
Effects of SFW on nuclear translocation of NF-*κ*B p65 through suppression of I*κ*Bα phosphorylation in LPS-stimulated BV-2 microglia. BV-2 cells were pretreated with SFW (200 μg/mL) for 1 h prior to stimulation with LPS (200 ng/mL) for 30 min. Cell lysates were prepared and analyzed by Western blotting with anti-phospho-I*κ*Bα antibody (**A**,**B**). Results are representative of those obtained from three independent experiments. ^###^ *p* < 0.001 versus control. *** *p* < 0.001 versus LPS. Localization of NF-*κ*B p65 was visualized with fluorescence microscopy after immunofluorescence staining with NF-*κ*B p65 antibody (red fluorescence) (**C**). Cells were stained with DAPI for visualization of nuclei (blue fluorescence). Scale bar: 50 μm. Arrows: highlight areas of cells with a translocation of NF-*κ*B p65.

We examined whether SFW had any impact on I*κ*Bα phosphorylation and the nuclear translocation of NF-*ĸ*B in LPS-stimulated BV-2 cells. As shown in Figure 5A,B, the LPS-treated group showed increased I*κ*B*α* phosphorylation, which was attenuated by SFW in a dose-dependent manner. Next, the inhibitory effect of SFW on NF-*ĸ*B activation was confirmed using immunocytochemistry. The LPS treatment also significantly enhanced the translocation of the NF-*ĸ*B p65 subunit into the nucleus, but the SFW treatment markedly blocked the translocation of NF-*ĸ*B into the nucleus (Figure 5C). Taken together, our results show that SFW attenuated the LPS-induced nuclear translocation of NF-*ĸ*B by suppressing I*κ*B*α* phosphorylation.

### 2.6. HPLC Analysis of Caffeic Acid

**Figure 6:**
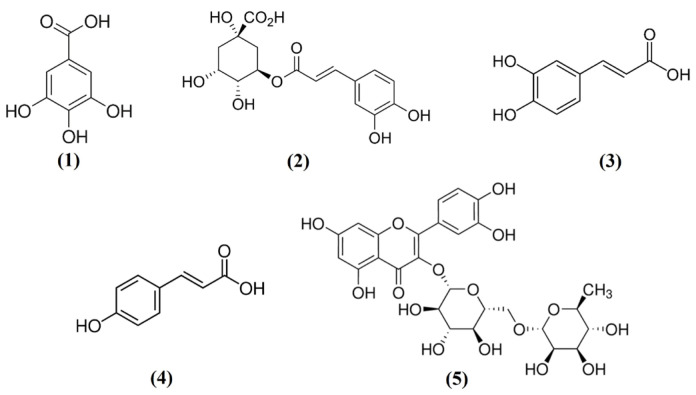
Chemical structure of gallic acid (**1**), chlorogenic acid (**2**), caffeic acid (**3**), *p*-coumaric acid (**4**), and rutin (**5**).

**Figure 7:**
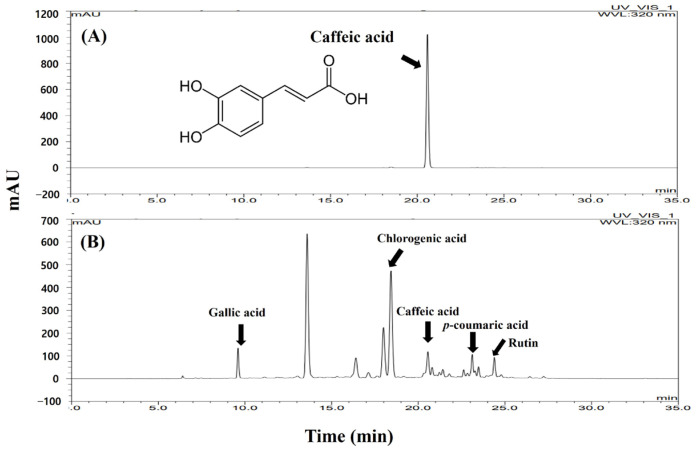
HPLC chromatogram of caffeic acid (**A**) and SFW (**B**). *X*-axis, retention time; *Y*-axis, absorbance unit. The monitoring wavelength was set at 320 nm.

The high-performance liquid chromatography (HPLC) chromatogram and the constituent compound chemical structures are shown in Figure 6. In this study, caffeic acid was detected in the SFW by HPLC-UV (Figure 7). For quantitative analyses, caffeic acid was used to analyze the linear calibration results (r^2^ = 0.99). The SFW contains 1.56 mg/g of caffeic acid. Additionally, we confirmed that SFW contained rutin, chlorogenic acid, and gallic acid as previously reported [19], as well as *p*-coumaric acid (Table 1).

However, comparing the peak sizes of SFW and SFE, we were most interested in caffeic acid, a component whose peak area was larger in SFW than in SFE (Figure S1). Therefore, we compared the content of caffeic acid and confirmed that the content in SFW (1.56 mg/g) was 3 times higher than that in SFE (0.55 mg/g). Although it has been shown that stems of *S. commixta* extract contain caffeic acid [20], this is the first to confirm that the fruits of *S. commixta* extract contain caffeic acid.

### 2.7. Anti-Inflammatory Effect of Caffeic Acid

**Figure 8:**
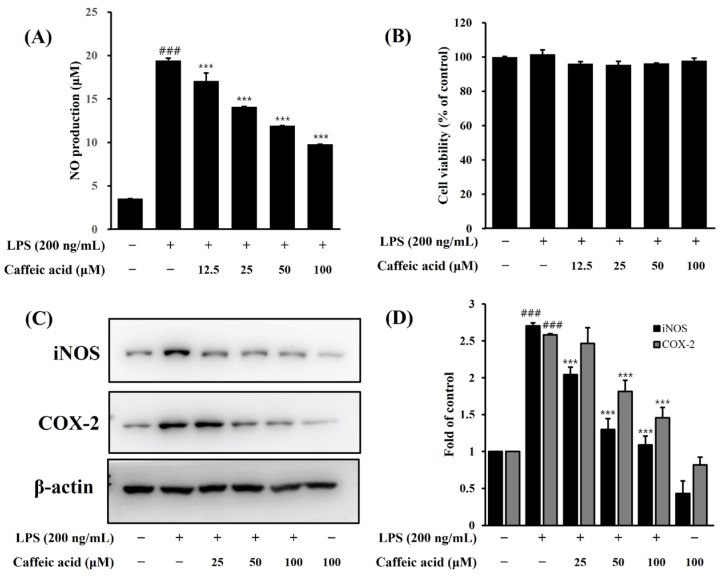
Effect of caffeic acid on NO production (**A**), cell viability (**B**) and iNOS and COX-2 protein expression (**C**,**D**) in LPS-induced BV-2 microglia. The BV-2 cells were pretreated with caffeic acid for 1 h, stimulated with LPS (200 ng/mL) for 18 h, and examined by Western blotting. Data are presented as mean ± SD from three independent experiments. β-actin was used as an internal control. ^###^ *p* < 0.001 versus control. *** *p* < 0.001 versus LPS.

We confirmed that caffeic acid (12.5–100 μM) has strong NO inhibitory activity without any toxicity on LPS-induced BV-2 cells (Figure 8A,B). It was observed that LPS treatment prominently increased NO production (19.4 ± 0.2 μM) in BV-2 cells compared to untreated cells, and this increase was markedly reduced by pretreatment with caffeic acid in a dose-dependent manner. Additionally, caffeic acid was found to dose-dependently inhibit the LPS-induced increase in iNOS and COX-2 protein expression (Figure 8C,D). Therefore, based on these results, we found that caffeic acid may be one of the main components of the anti-neuroinflammatory effect of SFW.

## 3. Discussion

Neurodegenerative diseases involve inflammation of the CNS [23], and neuroinflammation mediated by strongly activated microglia is involved in the pathology of various neurodegenerative disorders [24]. The overproduction of pro-inflammatory factors affects neuroinflammation and eventually leads to neurodegeneration and cell death [25]. Therefore, neuroinflammatory pathways have been recognized as potential therapeutic targets to prevent the progression of neurodegenerative diseases [26].

NO produced by the oxidation of nitrite plays an important role in the regulation of physiological synthesis and is mainly regulated by iNOS during inflammation [27]. When iNOS levels increase in response to pathogens, large amounts of NO are produced, resulting in the suppression of bacterial invasion and T cell proliferation, thereby alleviating local inflammatory responses [28]. However, when inflammation is not controlled, excessive overexpression of iNOS leads to excessive production of NO, ultimately resulting in cell damage and inflammation.

In the present study, we evaluated the effects of the SFW on pro-inflammatory factors such as NO, IL-6, and TNF-*α*. Our results showed that the SFW treatment significantly inhibited pro-inflammatory factors (Figure 1 and Figure 2) and suppressed LPS-stimulated iNOS and COX-2 expressions, which may have contributed to the inhibition of NO and other inflammatory factors (Figure 3).

LPS plays a central role in the inflammatory response, stimulating the production of inflammatory factors such as nitrites, prostaglandin E2, and leukotrienes and activating signaling pathways for these inflammatory factors [29]. LPS is generally known to activate MyD88 and TRIF signaling pathways in microglial cells [30]. The MyD88 pathway demonstrates a marked association with the NF-*κ*B and MAPK proteins, which play an important role in the expression of pro-inflammatory enzymes and cytokines related to inflammatory processes [30]. LPS also activates the MAPK pathway, another major extracellular signaling pathway stimulated by inflammatory factors [31].

MAPKs, a family of serine/threonine protein kinases including ERK, JNK, and p38, play an important role in regulating signaling involved in the production of neuroinflammatory factors [32,33,34]. Therefore, the inhibition of the NF-*κ*B and MAPK signaling pathways in activated microglia might be associated with an anti-inflammatory effect against LPS-stimulated inflammation. In our experiments, LPS has been shown to increase the phosphorylation of ERK, p38, and JNK, and pretreatment with SFW decreased the phosphorylation of p38, JNK, and ERK (Figure 4). Our findings are consistent with previous reports showing that anti-inflammatory potent substances inhibit pro-inflammatory mediators such as IL-6, TNF-*α*, and NO through the phosphorylation of MAPKs.

NF-*κ*B is an important transcription factor related to immune responses and plays a significant role in the induction of pro-inflammatory cytokine expression [35]. Under normal conditions, p65/p50 proteins are present in the cytoplasm of cells through binding to the I*κ*B protein and remaining inactive [36]. During inflammatory conditions induced by LPS, NF-*κ*B is activated through phosphorylation by I*κ*B kinase and the degradation of I*κ*B [34]. Previous studies have shown that the abnormal regulation of the NF-*κ*B pathway in microglia is involved in the development of pathological conditions such as ischemia, Alzheimer’s disease, and autoimmune encephalomyelitis [37]. Collectively, our results demonstrated that SFW attenuates the LPS-induced nuclear translocation of NF-*κ*B and the MAPK pathway.

In the present study, pretreatment with SFW suppressed the LPS-induced translocation of p65 into the nucleus through inhibiting the phosphorylation of I*κ*B (Figure 5A,B). In addition, the nuclear translocation of p65, a subunit of NF-*κ*B, triggered by LPS exposure, was also clearly blocked by the SFW (Figure 5C). Therefore, our data showed that the inhibitory effect of SFW on neuroinflammatory responses was mediated by inactivating the NF-*κ*B pathway.

Phytochemical analysis of the two extracts (SFW and SFE) through HPLC analysis showed that most major components of these extracts had similar retention times but also had different components (Suppl. S1). Among the ingredients in the SFW, we focused on caffeic acid as an anti-inflammatory ingredient. Moreover, this study reported for the first time that the fruit extract of *S. commixta* contains caffeic acid.

A previous study reported that caffeic acid has a wide range of beneficial effects such as antioxidant, anti-inflammatory, antibacterial, and antiviral effects [38]. The results of the present study showed that caffeic acid dose-dependently decreased NO production and inhibited the expression of iNOS and COX-2 in LPS-induced BV-2 cells at noncytotoxic concentrations. Therefore, we determined that caffeic acid plays a crucial role in the anti-neuroinflammatory effect of SFW.

To the best of our knowledge, the present study is the first to show that SFW effectively suppresses neuroinflammation, suggesting that SFW may have beneficial effects on various neurodegenerative diseases associated with neuroinflammation and microglial activation. Additional research is warranted to investigate animal experimentation and the other main ingredients of SFW.

## 4. Materials and Methods

### 4.1. Materials

Dulbecco’s modified Eagle’s medium (DMEM), fetal bovine serum (FBS), penicillin, and streptomycin were purchased from Gibco (Grand Island, NY, USA). LPS (*Escherichia coli* 055:B5), 3-(4,5-dimethylthiazol-2-yl)-2,5-diphenyltetrazolium bromide (MTT), and caffeic acid were obtained from Sigma Chemical Co. (St. Louis, MO, USA). ELISA kits for IL-6 and TNF-*α* were purchased from BD Biosciences (Franklin Lakes, NJ, USA). Antibodies against iNOS, COX-2, p38, phosphor (p)-p38, ERK1/2, and p-ERK1/2 were purchased from Santa Cruz Biotechnology Inc. (Dallas, TX, USA). Antibodies for JNK, p-JNK, beta-actin, I*κ*B-*α*, p-I*κ*B-α, and NF-*κ*B p65 were purchased from Cell Signaling Technology (Danvers, MA, USA). Polyvinylidene fluoride (PVDF) membrane was purchased from GE Healthcare Life Sciences (Amersham Hybond-P, Buckinghamshire, UK). All other chemicals were purchased from Sigma Chemical Co. (St. Louis, MO, USA).

### 4.2. Plant Materials and Sample Preparation

*Sorbus commixta* fruits were purchased from a herbal medicine market (Jecheon, Korea). The plant samples were authenticated by Prof. K. H. Leem (Department of Herbology, Semyung University, Jecheon-si, Republic of Korea), and the voucher specimen (SMU23-02) was deposited in the Semyung University Herbarium. The water extract (SFW) was filtered after boiling the sample for 2 h, and ethanol extract (SFE) was soaked in 70% ethanol twice for 24 h each time at room temperature. The extracts were then filtered and evaporated using a vacuum evaporator under reduced pressure and lyophilized to produce a dried powder extract.

### 4.3. Cell Culture and Cell Viability

BV-2 cells were cultured in DMEM containing 5% FBS, streptomycin (100 μg/mL), and penicillin (100 unit/mL) in a 5% CO_2_ incubator at 37 °C. The cytotoxicity of SFW and SFE on BV-2 cells was determined using an MTT assay. The BV-2 cells were seeded into 24-well plates at a concentration of 8.0 × 10^4^ cells/well. The next day, the cells were pretreated with extracts for 1 h, with or without LPS treatment (200 ng/mL), for 18–24 h. MTT stock solution (0.5 mg/mL) was added and incubated for 2 h. The supernatants were aspirated, and the formazan crystals were dissolved with dimethyl sulfoxide. The absorbance of the reaction mixtures was determined at 540 nm using a microplate reader. Cell viabilities were expressed as percentages of the control.

### 4.4. Nitric Oxide Assay

To investigate the effects of SFW and SFE on NO production in BV-2 cells, a Griess assay was performed. BV-2 cells were seeded into a 6-well culture plate at a density of 8 × 10^4^ cells/well. The next day, the cells were pretreated with various concentrations of SFW and SFE (12.5, 25, 50, 100, and 200 μg/mL) for 1 h, followed by LPS stimulation (200 ng/mL) for 18–24 h. The supernatant was collected for NO measurement using a Griess reagent. The absorbance of the reaction mixtures was determined at 540 nm using a microplate reader.

### 4.5. Enzyme-Linked Immunosorbent Assay (ELISA) for the Determination of Cytokine Levels

BV-2 cells were seeded into a 6-well culture plate (3.5 × 10^5^ cells/well). After 24 h, cells were pretreated with SFW for 1 h and incubated with LPS (200 ng/mL) for 18 h. The supernatant was collected, and the IL-6 and TNF-*α* levels were measured using an ELISA kit according to the manufacturer’s instructions.

### 4.6. Western Blot Analysis

After the SFW treatment in each experimental group, cells were harvested and lysed using lysis buffer. The lysates were incubated on ice for 15 min and centrifuged at 13,000 rpm for 15 min at 4 °C, and the protein fraction was collected. The protein concentration was determined using a Bradford assay. Proteins (15 μg) were separated by sodium dodecyl sulfate-polyacrylamide gel electrophoresis (SDS-PAGE) and transferred onto PVDF membranes for 2 h at 100 V. After blocking with 5% non-fat milk in TBS-T for 1 h, the membrane was washed and then incubated overnight with primary antibodies. The next day, the membrane was washed with TBS-T buffer three times for 10 min and incubated with horseradish peroxidase-coupled secondary antibodies for 1 h. The membrane was washed with TBS-T buffer three times for 10 min each. The protein bands were detected with enhanced chemiluminescence (ECL). β-actin was used as an internal reference.

### 4.7. Immunocytochemistry Staining

To observe the localization of NF-*κ*B in LPS-simulated BV-2 cells, the cells were pretreated with SFW for 1 h then treated with LPS (200 ng/mL) for 30 min. After washing three times with phosphate-buffered saline (PBS), the cells were fixed with 4% paraformaldehyde for 15 min and washed with PBS three times, and then incubated with primary antibody in 1% bovine serum albumin solution overnight at 4 °C. The next day, the cells were washed with PBS three times and incubated with secondary antibodies in 4′,6-diamino-2-phenylindole (DAPI)-containing solution for 1 h at room temperature. The cells were mounted, and then images were captured using a fluorescence microscope (Olympus, Japan).

### 4.8. HPLC Analysis

The standard compounds of caffeic acid (C0625, 98%), chlorogenic acid (C3878, 95%), gallic acid (G7384, 97.5–102.5%), *p*-coumaric acid (C9008, 98%), and rutin hydrate (R5143, 94%) were obtained from Sigma Chemical Co. (St. Louis, MO, USA). A Thermo UltiMate 3000 HPLC system comprising a vacuum degasser, quaternary pump, and UV detector was used. The column, a Shimadzu Shim-pack 5 μm C_18_, (250 × 4.6 mm) was maintained at 30 °C. The solvents used for separation were 0.2% phosphoric acid in water (*v*/*v*) (eluent A) and methanol (*v*/*v*) (eluent B). The gradient programming used was as follows: 0–5 min, linear gradient from 0% to 20% B; 5–15 min, linear gradient from 20% to 35% B; 15–20 min, linear gradient from 35% to 60% B; 20–30 min, linear gradient from 60% to 75% B; and 30–35 min, linear gradient from 75% to 100% B. The flow rate was 0.8 mL/min, the detection wavelength was 320 nm, and the sample injection volume was 20 μL. The chromatographic peaks of caffeic acid were confirmed by comparing their retention times and UV spectra with those of their reference standards. The working standard solution was injected into the HPLC chromatogram to obtain the peak area response. A standard graph was prepared by plotting the concentration versus area. Qualification was performed from the peak area of the sample using the standard graph.

### 4.9. Statistical Analysis

All experiments were carried out in triplicate. Statistical analysis was conducted with one-way ANOVA followed by Tukey’s multiple-comparison test post hoc analysis using GraphPad Prism (version 5, Dotmatics; La Jolla, CA, USA). Statistical significance was set at *p* < 0.05.

## 5. Conclusions

The present study is the first to show that SFW has anti-neuroinflammatory activities. SFW significantly inhibited the protein expression of iNOS, COX-2, and NO production in LPS-induced BV-2 microglial cells. SFW inhibited inflammation-associated pro-inflammatory factors by suppressing the NF-*κ*B and MAPK pathways. In addition, we assumed that caffeic acid is one of the main components of SFW and analyzed its neuroinflammatory effects. However, we believe that in addition to caffeic acid, there are other active substances in SFW that have neuroinflammatory effects, and we plan to reveal them through further study. According to our findings, SFW may be a promising therapeutic candidate for the prevention of various neurodegenerative diseases through suppressing inflammation associated with microglial activation. However, future studies should be conducted to confirm the mechanisms involved in the neuroinflammation inhibitory effect using animal models.

## Figures and Tables

**Table 1:** Retention time, formular, molecular weight, and CAS number of standard compounds.

	Compound	Retention Time (min)	Formular	Molecular Weight	CAS Number
1	Gallic acid	9.60	C_7_H_6_O_5_	170.12	149-91-7
2	Chlorogenic acid	18.45	C_16_H_18_O_9_	354.31	327-97-9
3	Caffeic acid	20.58	C_9_H_8_O_4_	180.16	331-39-5
4	*p*-Coumaric acid	23.03	C_9_H_8_O_3_	164.16	501-98-4
5	Rutin hydrate	24.17	C_27_H_30_O_16_ × H_2_O	610.52	207671-50-9

## Data Availability

Data are contained within the article and Supplementary Materials.

## Supplementary Materials

The following supporting information can be downloaded at https://www.mdpi.com/article/10.3390/molecules29235592/s1: Figure S1: HPLC chromatograms of caffeic acid as standard (A), SFW (B) and SFE (C).
