# Supplementary figures and images for: Sorbus commixta Fruit Extract Suppresses Lipopolysaccharide-Induced Neuroinflammation in BV-2 Microglia Cells via the MAPK and NF-κB Signaling Pathways
Source: Molecules. 2024 Nov 26;29(23):5592. doi: 10.3390/molecules29235592 (PMC11643002; doi:10.3390/molecules29235592)

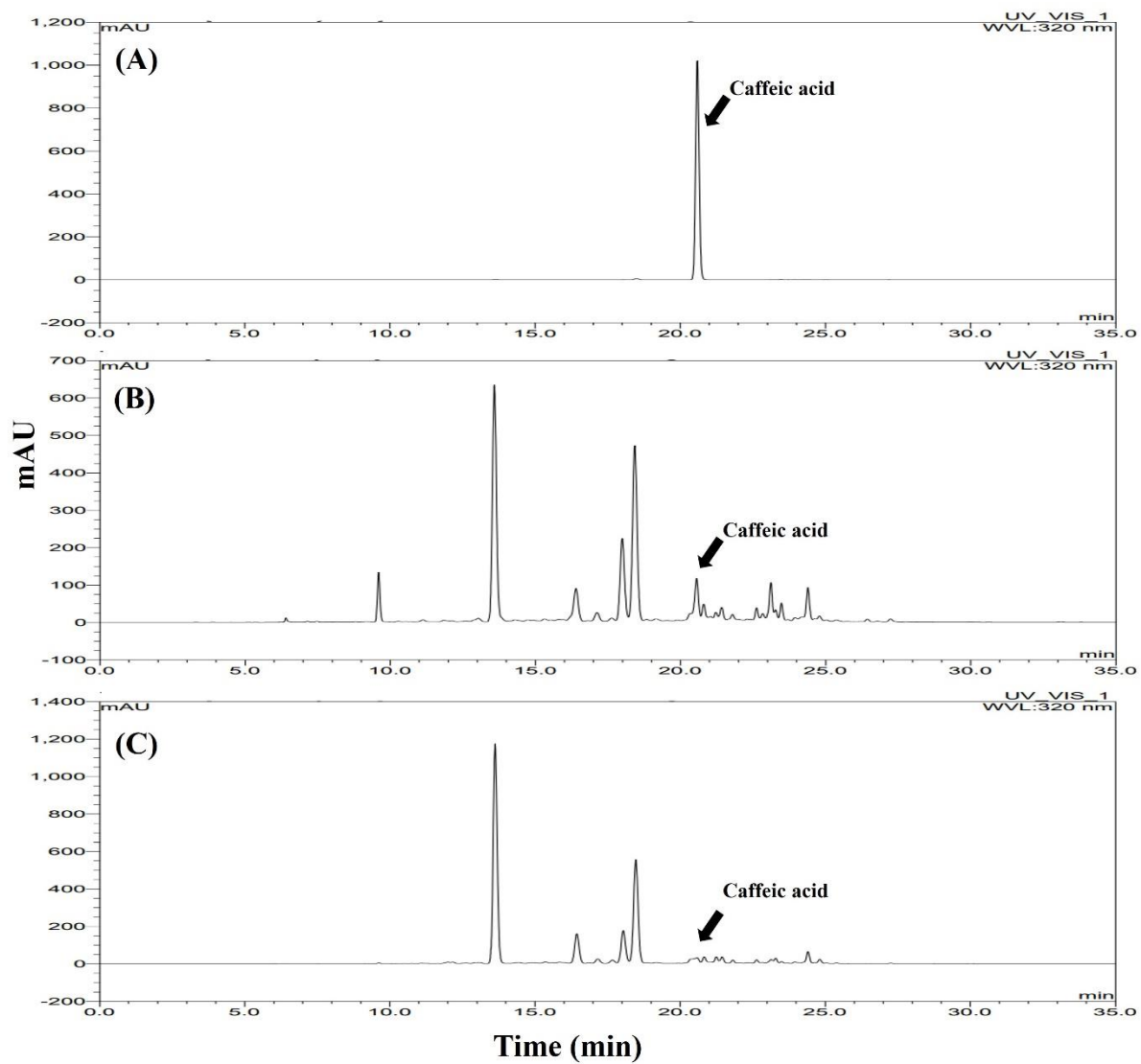

**Figure S1.** HPLC chromatograms of caffeic acid as standard (A), SFW (B) and SFE (C).

Supplement: Supplementary file 1 [file molecules-29-05592-s001.zip › molecules-3303843-supplementary.pdf]
